# In Vivo Measurements of Tumor Metabolism and Growth after Administration of Enzastaurin Using Small Animal FDG Positron Emission Tomography

**DOI:** 10.1155/2009/596560

**Published:** 2009-05-27

**Authors:** Karen E. Pollok, Michael Lahn, Nathan Enas, Ann McNulty, Jeremy Graff, Shanbao Cai, Jennifer R. Hartwell, Aaron Ernstberger, Donald Thornton, Les Brail, Gary Hutchins

**Affiliations:** ^1^Section of Pediatric Hematology & Oncology, Herman B Wells Center for Pediatric Research, Indiana University Simon Cancer Center, 1044 West Walnut Street R4 321, Indianapolis, IN 46202, USA; ^2^Therapeutic Area Oncology, Lilly Research Laboratories, Indianapolis, IN 46285, USA; ^3^Program Phase Statistics, Lilly Research Laboratories, Indianapolis, IN 46285, USA; ^4^Oncology Discovery, Lilly Research Laboratories, Indianapolis, IN 46285, USA; ^5^Department of Radiology, Indiana University Cancer Center, Indianapolis, IN 46202, USA

## Abstract

*Background*. The use of 2-[^18^F]fluoro-2-deoxy-D-glucose ([^18^F]FDG) may help to establish the antitumor activity of enzastaurin, a novel protein kinase C-beta II (PKC-*β*II) inhibitor, in mouse xenografts. *Methods*. The hematologic cell line RAJI and the solid tumor cell line U87MG were each implanted in NOD/SCID mice. Standard tumor growth measurements and [^18^F]FDG PET imaging were performed weekly for up to three weeks after tumor implantation and growth. *Results*. Concomitant with caliper measurements, [^18^F]FDG PET imaging was performed to monitor glucose metabolism. Heterogeneity of glucose uptake in various areas of the tumors was observed after vehicle or enzastaurin treatment. This heterogeneity may limit the use of [^18^F]FDG PET imaging to measure enzastaurin-associated changes in xenograft tumors. *Conclusion*. [^18^F]FDG PET imaging technique does not correlate with standard caliper assessments in xenografts to assess the antitumor activity of enzastaurin. Future studies are needed to determine the use of [^18^F]FDG PET imaging in preclinical models.

## 1. Introduction

Imaging techniques play an important role in the diagnosis, staging, and follow-up of cancer patients. While standard imaging techniques, such as computed tomography (CT), are based on differences in the anatomical structure of the tissues, positron emission tomography (PET) uses radiolabeled molecular probes to assess differences in biological or biochemical properties of tissues [1]. The most commonly used tracer for PET is the glucose analogue 2-[^18^F]fluoro-2-deoxy-D-glucose ([^18^F]FDG), which provides an estimate of tissue glucose utilization. Today, [^18^F]FDG PET is widely used in clinical diagnoses of cancer, including lung cancer [2], non-Hodgkin lymphoma [3], and glioblastoma [4]. Because of this increased utility in oncology [5–7], [^18^F]FDG PET is being evaluated as a tool to assess antitumor effects of standard or novel anticancer drugs in both human subjects and in animal models of cancer [8]. PET imaging may provide evidence of biological responses of novel anticancer compounds, which, in turn, can facilitate the transition of compounds from preclinical to clinical investigation. One such novel compound is enzastaurin, which was developed as a Protein kinase C-beta (PKC-*β*) inhibitor.

The family of Protein Kinase C (PKC) has been implicated in processes that control tumor growth, survival, and progression [9]. In particular, PKC-*β* activation has been recognized as an important contributor to malignant growth in diffuse large cell B cell lymphoma [10] and in glioblastoma [11]. Recently, enzastaurin has shown antitumor activity in xenograft models of the colorectal cancer cell line HCT116 and in the glioblastoma cell line U87MG [12]. While enzastaurin was designed as a selective PKC-*β* inhibitor, recent studies suggest that its antitumor activity is modulated by activation of GSK-3*β* and the PI3K/AKT pathway [12]. In this study we evaluated the extent to which [^18^F]FDG PET imaging can accurately characterize the antitumor activity of enzastaurin in two different mouse xenograft tumor models.

## 2. Material and Methods

### 2.1. Animal Model

Nonobese diabetic/severe combined immunodeficient (NOD/SCID) mice were raised and cared for by the Indiana University Simon Cancer Center Transplant and Xenograft Core following the institutional guidelines of animal care. The study was conducted from October 2004 to July 2005.

### 2.2. Cell Lines

The glioblastoma cell line, U87MG, and Burkitt's lymphoma cell line, RAJI, were obtained from the American Type Culture Collection (ATCC, Manassas, Va, USA) and cultured as recommended by ATCC. As previously published, U87MG and RAJI cells express high levels of PKC-*β* [12, 13].

### 2.3. Xenograft Tumor Studies

Prior to subcutaneous (s.c.) injection, cells were resuspended in a 1 : 1 ratio of tumor cells : matrigel (BD Biosciences, Bedford, Mass, USA) and Burkitt's lymphoma cell line, RAJI was resuspended in a 1 : 2 ratio of tumor cells : matrigel. Each mouse was injected s.c. in the right flank with 5 × 10^6^ cells. Mice were monitored daily for palpable tumors.

### 2.4. Enzastaurin Administration

Enzastaurin treatment was initiated when the tumors reached a volume of at least 150 mm^3^. Mice with similar tumor sizes were matched in the control and enzastaurin treated groups. Enzastaurin was suspended in 10% acacia (Fisher Scientific, Fair Lawn, NJ, USA) in water and dosed by gavage twice daily at 75 mg/kg based upon weekly body measurements for each treated group. Control groups were treated only with vehicle.

### 2.5. PET and CT Imaging

Each animal was anesthetized with acepromazine (1-2 mg/kg i.m.) and torbugesic (2 mg/kg i.m.) and placed on a custom bed for imaging. Animals were administered 0.5–1 mCi of [^18^F]FDG via a tail vein injection. A static 15-minute PET study was performed using the IndyPET II scanner [14, 15] at 45 minutes posttracer injection. Following the PET study, the animal bed was moved to and mounted on an EVS RS9 microCT scanner, and a volumetric image that encompassed the tumor volume was imaged at approximately 90 micron spatial resolution.

**FDG Utilization Estimates**

FDG uptake estimates are generated by placing ROIs on PET images acquired over the time period from 45–60 minutes posttracer administration. Indices of tumor FDG uptake were generated by calculating a ratio of the tumor to muscle uptake and by calculating the standardized uptake value (SUV):

(1) $${\text{SUV}}_{\text{FDG}}=\frac{\left[\int_{45}^{60}C_{t}\left(t\right)dt\right]}{\left(\rho \times ID\right)/M}.$$

In (1) *C*_*t*_(*t*) is the concentration of [F-18] in the tumor from the PET image, *M* is the mass of the animal, *ID* is the dose of the tracer injected into the animal, and *ρ* is tissue density. [^18^F]FDG was prepared by the Hamacher method using a commercial synthesis unit provided by PETNET/CTI (Knoxville, Tenn, USA) [16].

### 2.6. Statistical Analyses

Endpoints included tumor volume via calipers, tumor volume via CT, PET-determined measures for tumor, muscle, tumor/muscle ratio, and a standardized measure of uptake. Changes from baseline for each endpoint were analyzed for each cell line (U87MG, RAJI) and each time point (Week 2, Week 3) using nonparametric statistical methods (Wilcoxon rank-sum test) to compare treated versus control animals. Due to the exploratory nature of the analyses, *P*-values were used to indicate trends and potential future research hypotheses, rather than to test and confirm prespecified hypotheses.

## 3. Results

**Figure 1:**
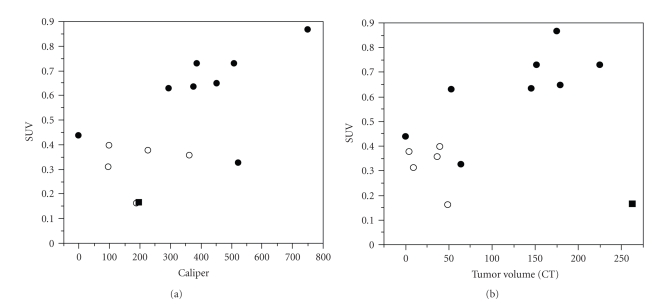
*Correlation of Standard Caliper Measurements to FDG-PET uptake measured by SUV (Panel (a)) and Correlation of SUV with CT-based Tumor Size (Panel (b))*: U87MG xenograft tumor cell growth was assessed over a period of 3 weeks using standard caliper measurements (mm^3^), serial FDG-PET uptake, and tumor size by CT scan (see Material and Methods). Correlation between the three different tumor growth measurements was plotted after 2 weeks (open symbols) and after 3 weeks (closed symbols) of tumor growth. Correlation between standard caliper measurements and standardized uptake value (SUV) was plotted (Panel (a)), and SUV was correlated with tumor size determined by CT scan (Panel (b)) (correlation coefficient 0.8, *P* = .001). (Correlation excludes one mouse with necrotic intratumoral tissue at Week 3 indicated by *■*).

We first investigated whether two tumor cell lines provided acceptable tumor growth in NOD/SCID mice to allow reproducible imaging with [^18^F]FDG PET (Figure 1). We used U87MG cells as a representative cell line for solid tumors, and RAJI as a model for hematologic cancers. Both the U87MG (Figure 1(a)) and the RAJI (data not shown) tumor types grew consistently in NOD/SCID mice prior to drug treatment and [^18^F]FDG PET imaging reliably detected glucose uptake in xenografts as determined by Standardized Uptake Value (SUV) (see Materials and Methods). In these feasibility studies, we also found that SUV correlated with U87MG tumor size as measured by CT (Figure 1(b)). Based on these preliminary observations of initial tumor growth, we elected to use both tumor cell lines to evaluate enzastaurin-induced metabolic changes as detected by [^18^F]FDG uptake. 

**Figure 2:**
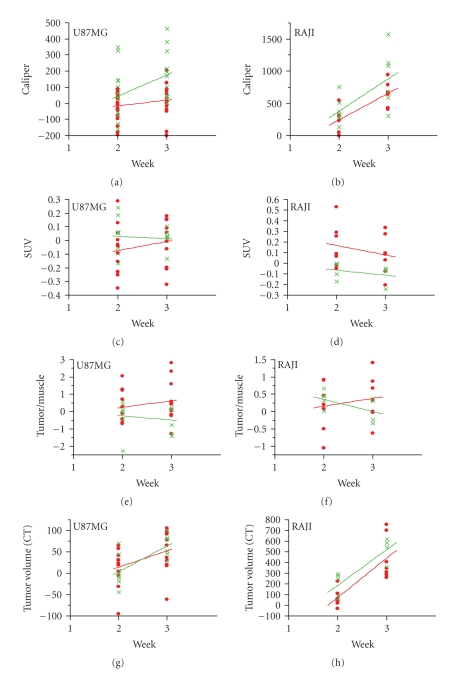
*Tumor assessments of Xenografts U87MG and RAJI at 2 and 3 weeks after enzastaurin treatment.* Standard caliper measurements show a tumor growth delay for U87MG (*P* < .05 at week 3), but not for RAJI (Panels (a) and (b)). [^18^F]FDG-PET imaging was performed at the same time as standard caliper measurements (Panels (c)-(d)). Tumor glucose metabolism changes were measured by SUV (Panels (c), (d)) and tumor/muscle ratio (Panels (e), (f)) in U87MG and RAJI xenografts. Only in RAJI xenografts enzastaurin treatment showed a trend in increased SUV (Panel (d); *P* < .10 at weeks 2 and 3). Using tumor/muscle ratio indicated a trend for FDG uptake in U87MG (*P* < .10 at week 3) (Panel (e)), but not in RAJI xenografts (Panel (f)). The tumor size assessment based on CT scan (Panels (g) and (h)) indicated a trend for detecting reduced tumor size only in RAJI xenografts after enzastaurin treatment at week 2 (*P* < .10). Mice treated with vehicle alone are shown in green; mice treated with enzastaurin are shown in red, representation of 2 independent experiments.

**Figure 3:**
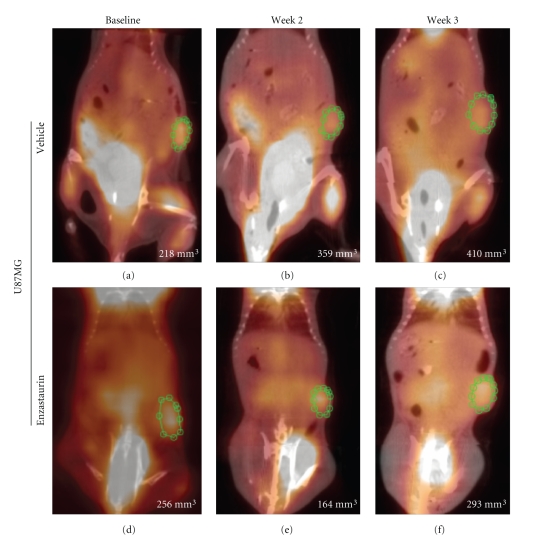
*SUV in NOD/SCID mice implanted with U87MG. * Representative mouse treated with vehicle (Panel (a)–(c)) and enzastaurin (Panel (d)–(f)) at baseline ((a), (d)) and after 2 weeks ((b), (e)) and 3 weeks ((c), (f)) of treatment. Green circle represents the region of interest (ROI) to assess changes in FDG uptake. Pictures are a fused image of the CT and PET. Each panel contains the caliper measurements (bottom right).

**Figure 4:**
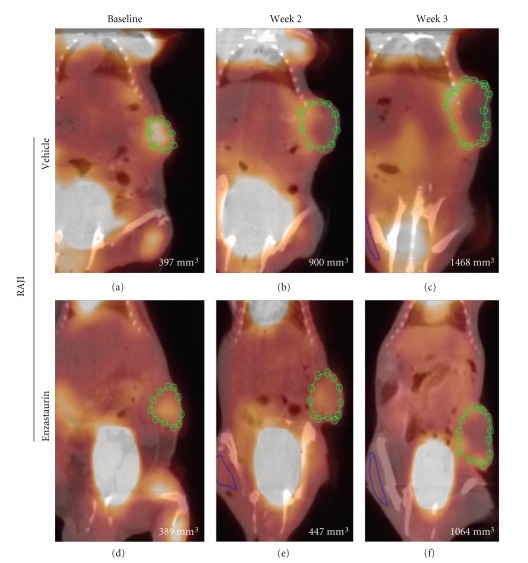
*SUV in NOD/SCID mice implanted with RAJI.* Representative mouse treated with vehicle (Panel (a)–(c)) and enzastaurin (Panel (d)–(f)) at baseline ((a), (d)) and after 2 weeks ((b), (e)) and 3 weeks ((c), (f)) of treatment. Green circle represents the region of interest (ROI) to assess changes in FDG uptake. Pictures are a fused image of the CT and PET. Each panel contains the caliper measurements (bottom right).

**Figure 5:**
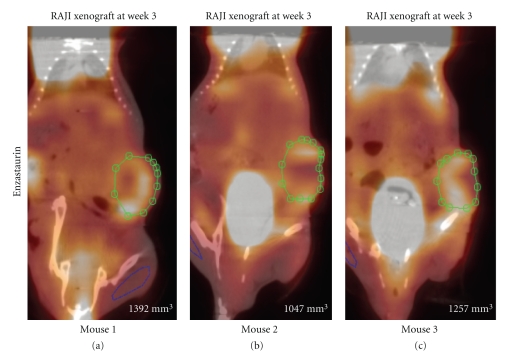
*SUV in NOD/SCID mice implanted with RAJI*. Representative 3 mice treated with enzastaurin for 3 weeks (Panel (a)–(c)). Green circle represents the region of interest (ROI) to assess changes in FDG uptake. Pictures are a fused image of the CT and PET. Each panel contains the caliper measurements (bottom right).

Enzastaurin-induced metabolic changes were evaluated using [^18^F]FDG uptake in two independent experiments for each tumor cell line, U87MG and RAJI, respectively (Table 1). Consistent with previous studies in nude mice [12], enzastaurin induced tumor growth delay in NOD/SCID mice implanted with U87MG and RAJI (Figures 2(a) and 2(b)). U87MG cells grew slower than RAJI cells, and enzastaurin had a tumor growth delay mainly in the U87MG tumor cell model (Figures 2(a) and 2(b)). A significant tumor growth delay was seen in U87MG after treatment with enzastaurin over the period of 3 weeks (Figure 2(a)). In the RAJI xenograft model, there was a trend in the tumor growth delay, but not a statistically significant difference between vehicle and enzastaurin-treated groups (Figure 2(b)). Concomitant with caliper measurements, [^18^F]FDG PET was performed. [^18^F]FDG PET imaging was evaluated using SUV (Figures 2(c) and 2(d)) and tumor/muscle ratio (Figures 2(e) and 2(f)). Enzastaurin administration did not alter tumor glucose metabolism as measured by SUV changes in U87MG xenografts (Figure 2(c)). Compared to RAJI, the SUV changes in U87MG xenografts occurred at smaller signal intensities, and thus contributed to an overlap of SUV measurements between vehicle- and enzastaurin-treated mice (compare Figures 2(c) and 2(d)). In RAJI, SUV uptake appeared to be increased at weeks 2 and 3 in enzastaurin-treated compared to vehicle-treated mice (*P* < .10 at weeks 2 and 3; Figure 2(d)). It is possible that metabolic heterogeneity of the tumor glucose metabolism in different areas of the tumor may have caused this difference in SUV uptake. In addition to SUV, we also used tumor/muscle ratio to determine the metabolic effect of enzastaurin in tumors. The tumor/muscle ratio uses muscle tissue with its low metabolic rate to normalize the tumor tissue [^18^F]FDG uptake. Based on this analysis there was no clear evidence of a metabolic change induced by enzastaurin compared to vehicle treatment (Figures 2(e) and 2(f)). However, for U87MG there was a trend in FDG uptake in enzastaurin-treated mice (*P* < .10 at week 3), which was not observed in RAJI xenografts (Figures 2(e) and 2(f), resp.). Next, we determined tumor volume by using standard volumetric measurements based on CT. After enzastaurin treatment, we observed a trend in tumor size reduction for RAJI xenografts but not for U87MG xenografts at week 2 (*P* < .10) (Figures 2(g) and 2(h)). While the collective analysis of all animals from 2 independent experiments did not reveal a clear distinction between enzastaurin- and vehicle-treated animals, there were some animals that did show a metabolic change after enzastaurin treatment. In these cases we saw a decline only after two weeks of treatment that was essentially undetectable after the third week of treatment (Figures 3(a)–3(f) and Figures 4(a)–4(f)). The changes observed in U87MG were different from changes observed in RAJI. In U87MG xenografts, [^18^F]FDG uptake declined after the 2nd week of treatment. The RAJI tumors were fast growing and had large areas of necrotic tissue and some areas with newly increased [^18^F]FDG uptake (Figure 5). The growth pattern of these tumors is partly responsible for the intertumoral heterogeneity seen in this study and may contribute to the lack of detecting enzastaurin-induced changes in the tumor.

## 4. Discussion

In this study we used a specialized PET imaging approach which was developed to assess activity of anti-cancer agents in small animals [14]. Changes in [^18^F]FDG uptake were generally found to correlate with reduction in tumor size as determined by caliper measurements [17, 18]. Our study uses a metabolic kinase inhibitor to compare drug-mediated tumor growth reduction with metabolic alterations in vivo. While previous imaging studies evaluated anti-tumor activity of cytotoxic agents, it is not clear how [^18^F]FDG PET imaging can help determine antitumor activity of kinase inhibitors. For instance, imatinib activity was evaluated in mice with limited success [19]. In addition to its cost, the use of PET imaging may be limited due to the spatial resolution of current PET scanners [20]. Thus, few studies have been published which evaluate the use of small animal imaging in drug discovery. 

For the first time, we assessed the anti-tumor activity of the serine/threonine kinase inhibitor enzastaurin by [^18^F]FDG uptake in mice. Because we used NOD/SCID instead of conventional nude mice, we first confirmed that [^18^F]FDG PET images could reproducibly be obtained in xenografts of glioblastoma and lymphoma. This feasibility assessment was important to establish the tumor size at which [^18^F]FDG uptake is detectable in mice. Tumors had to be at least 150 mm^3^ in volume to be visualized by the scanner, and the best assessment was observed in tumors that were more than 400 mm^3^ (Figure 1). This is consistent with other studies which reported on the need of specific tumor sizes for scanner assessment [21]. The subsequent studies with enzastaurin treatment did not provide clear evidence of enzastaurin-induced metabolic changes in either of the two tumor types examined. However, there are several possibilities why enzastaurin-induced changes were not detected by [^18^F]FDG PET imaging. 

First, it is possible that enzastaurin may not have a homogenous impact on the metabolic rate in the tumor tissue. Although PKC isoenzymes have been implicated in cell proliferation [9], their specific role during glucose metabolism is still not understood. On one hand, glucose can induce PKC-*β* expression [22], and on the other hand, overexpression of PKC-*β* reduces glucose uptake in cells [23]. Hence, selective PKC-*β* inhibitors have been investigated as potential treatments for diabetes [24]. Whether such a PKC-*β*-dependent glucose regulation exists in tumor cells is not known [25]. Considering the observation of this study, in which tumor growth delay and glucose metabolism are not correlated with enzastaurin activity in xenograft tumors, it is possible that enzastaurin is not able to modulate the complex glucose regulation in the tumor cells [26]. Recently, the antiangiogenic kinase inhibitor AZD2171 was also evaluated in a small animal study for its metabolic change in tumors. Compared to [^18^F]FDG PET imaging, only [^18^F] fluoromethane proved as a useful tool to assess the anti-tumor activity of AZD2171 [27]. Therefore, it appears that only some kinase inhibitors will have metabolic changes in tumors, which can be assessed by [^18^F]FDG PET imaging.

Secondly, the s.c. implanted tumors tend to grow initially along the skin surface and infiltrate the underlying tissue to a lesser extent. This observation might explain why perhaps the caliper measurements can be used to demonstrate anti-tumor effects while the imaging of the deeper tissue by [^18^F]FDG or CT is not able to detect differences of treatment effect. Because of the inability to delineate clearly the borders of the infiltrating tumor tissue, the current study may underestimate the metabolic change and thus lead to false-negative imaging results. In some animals, we used contrast dye to delineate better the tumor border in mice. In these instances, caliper and CT scans showed comparable results. Hence, future studies will need to be conducted with contrast imaging techniques to approximate the anatomical borders of the tumor. Tumor weight did not correlate with caliper measurements, because CT scans or caliper measurements were taken during the course of the study, while tumor weight was collected only at the end of the study, when animals were sacrificed, and tumors had become heterogeneous.

Finally, the observed [^18^F]FDG uptake signals are considerably lower relative to what is measured in humans. The low baseline level in the present xenograft study negatively impacted the ability to assess early changes in metabolism associated with treatment effect. Other factors influencing the low uptake signal are heterogeneity of glucose uptake in tumors, possible emergence of drug-resistant tumor cells, variability of pharmacokinetic exposure of the inhibitor, and the diffusion of tracer to the tumor site. 

In summary, our data are consistent with the hypothesis that shrinking tumor size (assessed by caliper measurements) does not equate to reduction in overall tumor metabolism. In fact, “pockets” of drug-resistant tumor cells with increased glucose uptake and growth kinetics may become prevalent in certain areas of the tumor, while glucose reduction will be present in other areas. Additional longitudinal PET imaging studies will have to examine in detail the variables which affect the accurate measurement of tumor response to drug treatment. Our study implies that [^18^F]FDG PET imaging will be useful only in a select type of tumors and perhaps detect antitumor activity only for a limited number of drugs. 

## Figures and Tables

**Table 1:** Evaluable sample sizes for caliper measures (CT/PET measures in parentheses).

	U87MG (*n*)		RAJI (*n*)	
Week	Enzastaurin	Vehicle	Enzastaurin	Vehicle

2	19 (12)	15 (8)	7 (7)	7 (5)
3	15 (11)	12 (6)	7 (7)	6 (4)
